# Indoxyl sulfate, a uremic toxin, downregulates renal expression of Nrf2 through activation of NF-κB

**DOI:** 10.1186/1471-2369-14-56

**Published:** 2013-03-04

**Authors:** Dilinaer Bolati, Hidehisa Shimizu, Maimaiti Yisireyili, Fuyuhiko Nishijima, Toshimitsu Niwa

**Affiliations:** 1 Department of Advanced Medicine for Uremia, Nagoya University Graduate School of Medicine, 65 Tsurumai-cho, Showa-ku, Nagoya, Japan; 2 Biomedical Research laboratories, Kureha Co., Tokyo, Japan

**Keywords:** NF-κB, Heme oxygenase-1 (HO-1), NAD(P)H:quinone oxidoreductase 1 (NQO1), 8-hydroxydeoxyguanosine (8-OHdG), Proximal tubular cells

## Abstract

**Background:**

Indoxyl sulfate, a uremic toxin, is accumulated in the serum of chronic kidney disease (CKD) patients, accelerating the progression of CKD. In CKD rat kidney, the expressions of nuclear factor (erythroid-derived 2)-like 2 (Nrf2) and its related genes are downregulated. AST-120, an oral sorbent, reduces serum indoxyl sulfate and slows the progression of CKD. The present study aimed to determine whether indoxyl sulfate downregulates Nrf2 expression in human proximal tubular cells and rat kidneys and whether AST-120 upregulates Nrf2 expression in CKD rat kidneys.

**Methods:**

Effects of indoxyl sulfate on expression of Nrf2 were determined using HK-2 cells as human proximal tubular cells and the following animals: (1) Dahl salt-resistant normotensive rats (DN), (2) Dahl salt-resistant normotensive indoxyl sulfate-administered rats (DN+IS), (3) Dahl salt-sensitive hypertensive rats (DH), and (4) Dahl salt-sensitive hypertensive indoxyl sulfate-administered rats (DH+IS). Further, AST-120 was administered to subtotally nephrectomized CKD rats to determine its effect on the expression of Nrf2.

**Results:**

Indoxyl sulfate downregulated Nrf2 expression in HK-2 cells. The indoxyl sulfate-induced downregulation of Nrf2 expression was alleviated by an inhibitor of nuclear factor-κB (NF-κB) (pyrrolidine dithiocarbamate) and small interfering RNA specific to NF-κB p65. DN+IS, DH, and DH+IS rats showed decreased renal expression of Nrf2 and its downstream target genes, heme oxygenase-1 (HO-1) and NAD(P)H:quinone oxidoreductase 1 (NQO1), and increased renal expression of 8-hydroxydeoxyguanosine (8-OHdG), a marker of reactive oxygen species (ROS), compared with DN. Thus, indoxyl sulfate, as well as hypertension, downregulated renal expression of Nrf2 in rats. AST-120 upregulated renal expression of Nrf2, HO-1 and NQO1 and suppressed renal expression of 8-OHdG compared with control CKD rats.

**Conclusions:**

Indoxyl sulfate downregulates renal expression of Nrf2 through activation of NF-κB, followed by downregulation of HO-1 and NQO1 and increased production of ROS. Further, AST-120 upregulates renal expression of Nrf2 in CKD rats by removing serum indoxyl sulfate, followed by upregulation of HO-1 and NQO1 and decreased production of ROS.

## Background

Indoxyl sulfate, a uremic toxin, accelerates the progression of chronic kidney disease (CKD). Indoxyl sulfate is a metabolite of tryptophan in dietary proteins and is synthesized in the liver from indole that is produced by intestinal flora including *Escherichia coli*. Indoxyl sulfate is normally excreted into urine. As renal function deteriorates, however, indoxyl sulfate accumulates in serum due to its reduced renal clearance [1,2]. Accumulated indoxyl sulfate in serum is incorporated into the basolateral membrane of renal proximal tubular cells by mediating organic anion transporter types 1 and 3 [3]. Indoxyl sulfate induces cellular senescence and dysfunction of kidneys and proximal tubular cells [4-12]. Indoxyl sulfate induces reactive oxygen species (ROS) which activates nuclear factor-κB (NF-κB) and p53 [9-11]. Subsequently, indoxyl sulfate induces renal expression of fibrotic genes such as transforming growth factor-β1 (TGF-β1) [4,5] and α-smooth muscle actin (α-SMA) [9,10], inflammatory genes such as monocyte chemotactic protein-1 (MCP-1) [11], and cellular senescence [9,10], and suppresses renal expression of Klotho [8]. AST-120, an oral sorbent, decreases serum level of indoxyl sulfate by adsorbing indole, a precursor of indoxyl sulfate, in the intestine [13-17], and thereby suppresses progression of CKD.

Nuclear factor (erythroid-derived 2)-like 2 (Nrf2) is a basic leucine zipper transcription factor, and regulates induction of numerous antioxidant and phase II detoxifying enzymes [18]. Nrf2 is usually localized in the cytoplasm and associated with a repressor molecule known as Kelch-like ECH-associating protein 1 (Keap1), which facilitates its ubiquitination with subsequent degradation [19]. Keap1 contains several reactive cysteine residues that serve as sensors of intracellular redox state. There are two pools of Nrf2 proteins, free floating Nrf2 (fNrf2) and Keap1-binding Nrf2 (kNrf2). Nrf2 signaling is repressed by Keap1 at basal condition and induced by oxidative stress. Oxidative stress can impede Keap1-mediated Nrf2 ubiquitination but fail to disrupt Nrf2/Keap1 binding. When stimulated by oxidative stress, the ubiquitination and the proteasomal degradation are inhibited. Keap1 is saturated by undegradated kNrf2. However, protein translation is elevated. In combination, the pool of fNrf2 expands. fNrf2 can sense the change of redox milieu and transmit redox signals to cell nucleus via gradient nuclear translocation [19]. Nrf2 translocates to the nucleus and binds to an antioxidant response element (ARE), followed by transcriptional induction of ARE-bearing genes that encode antioxidative and detoxifying proteins such as heme oxygenase-1 (HO-1) and NAD(P)H:quinone oxidoreductase 1 (NQO1) [18]. The deletion of Nrf2 in murine models results in structural and functional kidney defects that are associated with increased inflammation and oxidative stress [18].

HO-1 catalyzes the first and rate limiting enzyme step of heme degradation and produces carbon monoxide (CO), iron and biliverdin, which is converted into bilirubin via biliverdin reductase. The expression of HO-1 is upregulated by a variety of oxidative stress stimuli as an adaptive cellular response against the toxicity of oxidative stress. The deletion of HO-1 in murine unilateral ureteral obstruction models promotes epithelial-mesenchymal transition, renal fibrosis, and apoptosis [20]. NQO1 catalyzes the oxidation of NAD(P)H to NAD(P)^+^ by various quinones. Activation of NQO1 leads to diminished renal damage through reduced oxidative stress by mediating NADPH oxidase activity via modulation of the cellular NAD(P)H:NAD(P)^+^ ratio in Dahl salt-sensitive rats on a high-salt diet [21].

The expressions of Nrf2 and its downstream target genes such as HO-1 and NQO1 are impaired in the kidneys of CKD rats [22]. The present study aimed to determine whether indoxyl sulfate is involved in downregulation of Nrf2 functions in kidneys, and how indoxyl sulfate regulates Nrf2 expression in proximal tubular cells. Furthermore, the present study aimed to determine whether AST-120 increases renal expression of Nrf2 in CKD rats.

## Methods

### Reagents

Reagents were obtained from the following suppliers: rabbit polyclonal anti-Nrf2 (sc-722) for immunohistochemistry and immunoblotting, Santa Cruz Biotechnology (Santa Cruz, CA, USA); rabbit polyclonal anti-HO-1 (ab13243) for immunohistochemistry, Abcam (Cambridge, UK); rabbit polyclonal anti-NQO1 (ab34173) for immunohistochemistry, Abcam (Cambridge, UK); mouse monoclonal anti-8-hydroxydeoxyguanosine (8-OHdG) (MOG-020P) for immunohistochemistry, Japan Institute for the Control of Aging, NIKKEN SEIL, (Shizuoka, Japan); anti-α-tubulin for immunoblotting, Calbiochem (La Jolla, CA, USA); anti-rabbit IgG horseradish peroxidase (HRP)-linked antibody, anti-mouse IgG, HRP-linked antibodies, Cell Signaling Technology (Beverly, MA, USA); indoxyl sulfate, Alfa Aesar (Lancashire, UK); pyrrolidine dithiocarbamate (PDTC) (an inhibitor of NF-κB), Sigma chemical (St. Louis, MO, USA); Dulbecco's modified Eagle's medium (DMEM)/F12, Wako (Osaka, Japan); trypsin-EDTA, fetal bovine serum (FBS), and insulin-transferrin-selenium, GIBCO (Grand Island, NY, USA); penicillin and streptomycin, Nacalai Tesque (Kyoto, Japan).

### Cell culture

HK-2 cells (human proximal tubular cells) purchased from ATCC (Manassas, VA, USA) were maintained in DMEM/F12 supplemented with 10% FBS, insulin (10 μg/ml insulin-transferrin (5.5 μg/ml)-selenium (6.7 ng/ml), 100 U/ml penicillin, and 100 μg/ml streptomycin.

### Preparation of Small Interfering RNAs Specific to NF-κB p65

Knockdown analysis was performed as described previously [10]. The sense sequence of the siRNA for NF-κB p65 was: 5’-AGAGGACAUUGAGGUGUAUdTdT-3’.

### Quantitative real time PCR

Quantitative real time PCR was performed as described previously [8-11]. Oligonucleotide primers were: human Nrf2, 5’-ACACGGTCCACAGCTCATC-3’ (forward) and 5’-TGTCAATCAAATCCATGTCCT-3’ (reverse); human GAPDH, 5’-ATGGGGAAGGTGAAGGTCG-3’ (forward) and 5’-GGGGTCATTGATGGCAACAATA-3’ (reverse). The expression of mRNA levels was measured as the ratio of each mRNA to GAPDH mRNA.

### Immunoblotting

Immunoblotting was performed as described previously [12]. Nrf2 and p65 were detected using specific antibodies and normalized to α-tubulin. The protein bands were visualized using the enhanced Chemi-Lumi One system (Nacalai Tesque).

### Animal study 1

Experimental rats were produced as reported previously [6]. Five-week-old Dahl salt-resistant rats (Dahl-Iwai, n = 16) and Dahl salt-sensitive rats (Dahl-Iwai, n = 16) were purchased from Japan SLC, Inc. (Hamamatsu, Shizuoka, Japan), and were fed powder rat chow (CE-2, Clea, Tokyo, Japan) and water for 2 weeks. Later, the rats were fed the chow (CE-2) with salt (1.5% NaCl) dissolved in the water. At 16 weeks of age, Dahl salt-sensitive rats spontaneously developed hypertension with systolic blood pressure (BP) of >140 mm Hg; and the rats were divided into the following 2 groups: Dahl salt-resistant normotensive rats (DN, n=16) and Dahl salt-sensitive hypertensive rats (DH, n = 16), and then rats were further randomized into 2 groups: control rats and indoxyl sulfate-administered rats (200 mg/kg/day of indoxyl sulfate in water). Therefore, the 4 groups were as follows: (1) Dahl salt-resistant normotensive rats (DN, n=8), (2) Dahl salt-resistant normotensive indoxyl sulfate-administered rats (DN + IS, n=8), (3) Dahl salt-sensitive hypertensive rats (DH, n=8), and (4) Dahl salt-sensitive hypertensive indoxyl sulfate-administered rats (DH + IS, n=8). At 48 weeks of age (32nd week of the study), kidney tissues were excised for immunohistochemical analysis. Serum indoxyl sulfate levels were measured by high-performance liquid chromatography [23,24].

### Animal study 2

Experimental rats were produced as reported previously [17]. Seven-week old male Sprague-Dawley rats (Clea, Tokyo, Japan) were used to produce CKD rats by 4/5-nephrectomy. In the first session, while the renal artery and vein of the left kidney were ligated, three-fifths of the left kidney was removed with a razor, and thrombin was applied onto the cut surface for hemostasis. Then, the artery and vein of the left kidney were unligated. One week after the first operation, the right kidney was removed after ligation of the renal artery and vein. These operations were performed under anesthesia with sodium pentobarbital (Schering-Plough, Corp., NJ, USA). Eleven weeks after subtotal nephrectomy, the rats were randomized into two groups, control CKD rats (n=9), and AST-120-treated CKD rats (n=9). AST-120 was orally administered to the rats at a dose of 4 g/kg/day with powder chow (CE-2, Clea) for 10 weeks, whereas powder chow alone was administered to control CKD rats. A control CKD rat died of uremia. Normal rats (n=9) were used to compare the data with CKD rats.

The Animal Care Committee of Kureha Biomedical Research Laboratories approved these animal studies (1 and 2), which proceeded according to the Guiding Principles for the Care and Use of Laboratory Animals of the Japanese Pharmacological Society.

### Immunohistochemistry

Immunohistochemistry of Nrf2, HO-1 and 8-OHdG was performed using the streptavidin-biotin complex (SABC) method as described previously [7,17]. Sections were incubated with the primary antibody (Nrf2, 1:100; HO-1, 1:50; NQO1, 1:100; 8-OHdG 1:20). Immunostaining positive areas were quantified in 10 random sections in the renal cortex using NIH Image 1.62.

### Statistical analysis

Data are expressed as mean±SE. The differences in immunohistochemical values were analyzed by Fisher’s least significant difference (LSD) test of ANOVA. To compare values between two groups, Student’s t test was used. Results were considered statistically significant when the *P* value was less than 0.05.

## Results

### Indoxyl sulfate downregulates Nrf2 expression in human proximal tubular cells through activation of NF-κB

**Figure 1:**
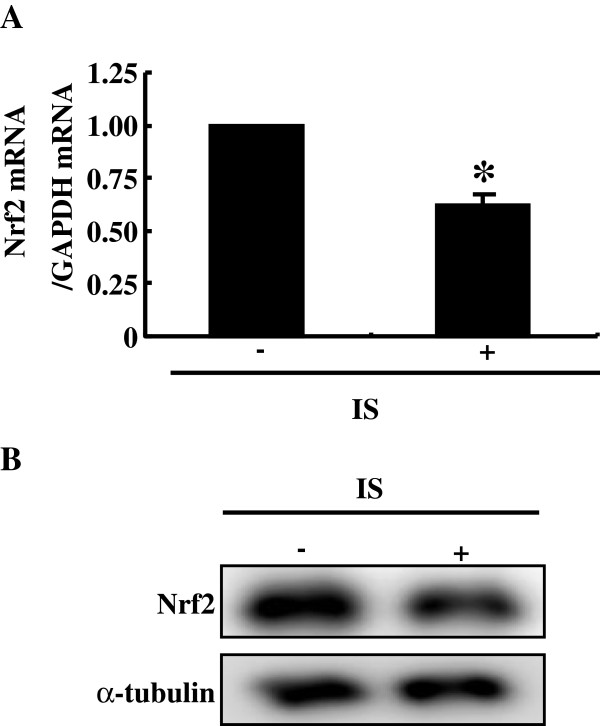
**Indoxyl sulfate decreases Nrf2 expression in HK-2 cells. A**: serum-starved HK-2 cells were incubated with or without indoxyl sulfate (250 μM) for 48 h. Expression levels of Nrf2 mRNA were measured by real-time PCR. Data are expressed as mean±SE (n=4). *P < 0.05 vs. untreated cells. **B**: experimental conditions were as described in *A* except treatment with indoxyl sulfate for 72 h was used in place of 48 h. Whole cell lysates were immunoblotted using anti-Nrf2 antibody.

HK-2 cells were cultured with or without indoxyl sulfate at a concentration of 250 μM which is comparable to its mean serum level in patients on hemodialysis [1]. Indoxyl sulfate-treated cells showed significantly reduced both mRNA and protein expressions of Nrf2 as compared with untreated cells (Figure 1). Thus, indoxyl sulfate decreased Nrf2 expression in HK-2 cells. Because the expression of Nrf2 protein in the whole cell lysates was reduced, the expressions of its downstream genes are considered to be decreased.

**Figure 2:**
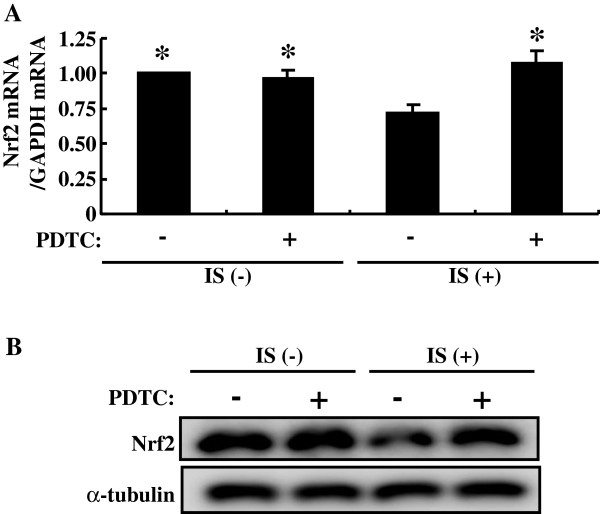
**An inhibitor of NF-κB alleviates indoxyl sulfate-induced decrease of Nrf2 expression in HK-2 cells. A**: serum-starved HK-2 cells were incubated with or without PDTC (10 μM) for 30 min, followed by indoxyl sulfate (250 μM) for 48 h. Expression levels of Nrf2 mRNA were measured by real-time PCR. Data are expressed as mean±SE (n=4). *P < 0.05 vs. indoxyl sulfate-treated cells without PDTC. **B**: experimental conditions were as described in *A* except treatment with indoxyl sulfate for 72 h was used in place of 48 h. Whole cell lysates were immunoblotted using anti-Nrf2 antibody.

**Figure 3:**
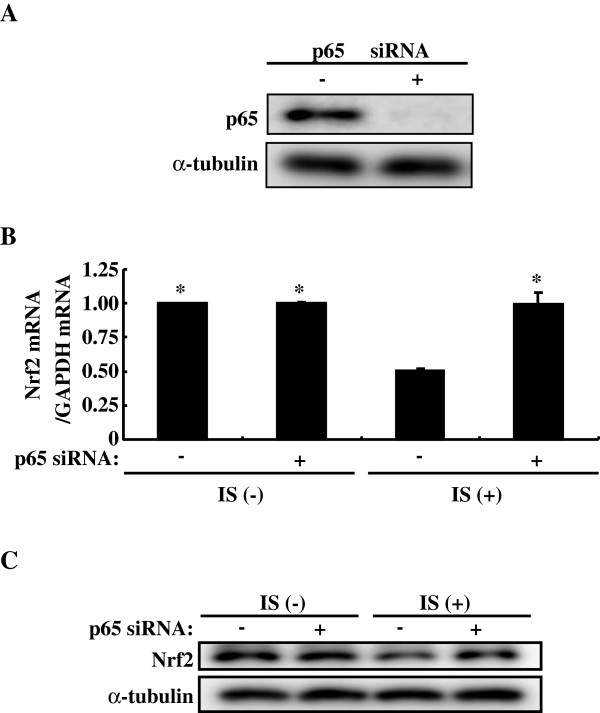
**Knockdown of p65 alleviates indoxyl sulfate-induced decrease of Nrf2 in HK-2 cells. A**: HK-2 cells were transfected with or without NF-κB p65 siRNA (10 nM), and then serum starved for 24 h. Whole cell lysates were immunoblotted using anti-Nrf2 antibody. **B**: HK-2 cells were transfected with or without NF-κB p65 siRNA (10 nM), and then serum starved for 24 h, followed by indoxyl sulfate (250 μM) for 48 h. Expression levels of Nrf2 mRNA were measured by real-time PCR. Data are expressed as mean±SE (n=4). *P < 0.05 vs. indoxyl sulfate-treated cells without p65 siRNA. **C**: experimental conditions were as described in B except treatment with indoxyl sulfate for 72 h was used in place of 48 h. Whole cell lysates were immunoblotted using anti-Nrf2 antibody.

Indoxyl sulfate activates NF-κB in HK-2 cells as reported previously [10]. In the present study, we used PDTC to examine relationship between NF-κB activation and Nrf2 expression. PDTC blocks degradation of IκB and thereby inhibit NF-κB activity. PDTC alleviated indoxyl sulfate-induced downregulation of both Nrf2 mRNA and protein expression (Figure 2). We further confirmed these results with NF-κB p65 siRNA. The expression of NF-κB p65 was reduced by its siRNA (Figure 3). Indoxyl sulfate-induced downregulation of both Nrf2 mRNA and protein expression was relieved by NF-κB p65 siRNA as well as by PDTC. Thus, indoxyl sulfate-induced activation of NF-κB downregulates Nrf2 expression in HK-2 cells.

### Indoxyl sulfate suppresses Nrf2 functions in rat kidneys

To confirm whether indoxyl sulfate reduces Nrf2 functions in the kidney, the effect of indoxyl sulfate on the expression of Nrf2 in the kidneys of normotensive and hypertensive rats was determined in animal study 1. Laboratory parameters of these rats including serum indoxyl sulfate (Table 1) were described previously [6]. Systolic BP at the 32nd weeks of the study were as follows; 143±3 mmHg in DN rats, 141±3 mmHg in DN+IS rats, 158±5 mmHg in DH rats, and 158±9 mmHg in DH+IS rats [6].

**Table 1:** Serum levels of indoxyl sulfate at the 32nd weeks of animal study 1 and at the 10th week of animal study 2

	**Serum indoxyl sulfate (mg/dl)**
Animal study 1	
DN	0.10±0.01
DN+IS	0.94±0.13**^###^
DH	0.06±0.01
DH+IS	1.89±0.26**^###^
Animal study 2	
Normal	0.08±0.007
CKD	0.52 ± 0.16*
CKD+AST-120	0.12±0.02#

Data are expressed as mean±SE (n=8 for animal study 1, or n=9 for animal study 2).

**P < 0.01 vs. DN; ^###^*P*<0.001 vs. DH.

* *P* <0.05 vs. normal, ^#^*P* <0.05 vs. CKD.

**Figure 4:**
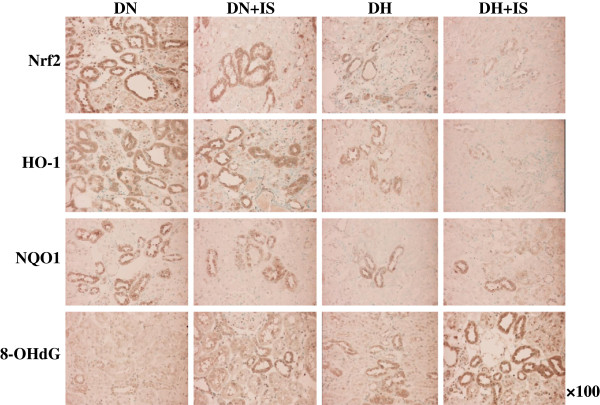
**Immunohistochemistry of Nrf2, HO-1, NQO1 and 8-OHdG in the kidneys of DN, DN+IS, DH, and DH+IS rats.** Nrf2, HO-1 and NQO1 were localized in the cytoplasm of tubular cells, especially in the kidneys of DN rats. 8-OHdG, a marker of ROS, was localized in the nuclei of tubular cells, especially in the kidneys of DH+IS rats.

**Figure 5:**
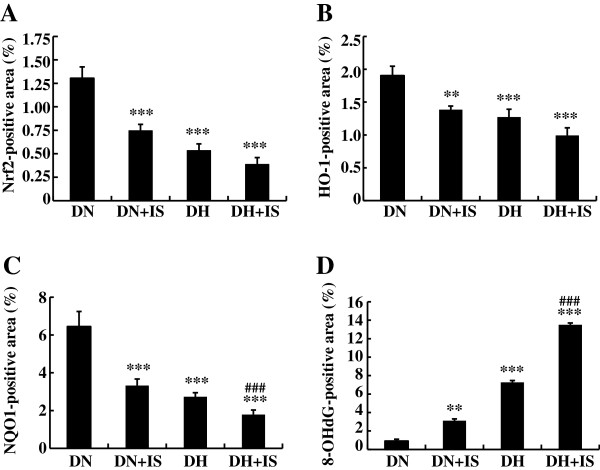
**Immunostaining-positive areas of Nrf2, HO-1, NQO1 and 8-OHdG in the kidneys of DN, DN+IS, DH, and DH+IS rats.** Immunostaining-positive areas of Nrf2, HO-1 and NQO1 were significantly decreased in DN+IS, DH and DH+IS rats as compared with DN rats. On the other hand, immunostaining-positive area of 8-OHdG was significantly increased in DN+IS, DH and DH+IS rats as compared with DN rats. Data are expressed as mean±SE (n=8). **P < 0.01, ***P < 0.001 vs. DN; ^###^*P*<0.001 vs. DH.

Expression levels of Nrf2, HO-1, and NQO1 were reduced in DN+IS, DH and DH+IS rats as compared with DN rats (Figures 4 and 5). On the other hand, staining level of 8-OHdG, a ROS maker, was increased in the kidneys of DN+IS, DH, and DH+IS rats as compared with DN rats (Figures 4 and 5). In addition, staining level of 8-OHdG in the kidneys of DH+IS rats was significantly high as compared with DH rats (Figures 4 and 5). Taken together, indoxyl sulfate, as well as hypertension, suppresses Nrf2 expression in the kidney, followed by decreased expression of HO-1 and NQO1 and increased level of 8-OHdG.

### AST-120 stimulates Nrf2 functions in the kidneys of CKD rats by reducing serum level of indoxyl sulfate

The preset study investigates whether indoxyl sulfate induces downregulation of Nrf2 expression in rat kidneys. To verify this hypothesis, the effect of AST-120 which reduces the serum level of indoxyl sulfate, on the expression of Nrf2 in the kidneys of CKD rats was determined in animal study 2. Laboratory parameters of these rats including serum indoxyl sulfate (Table 1) were described previously [17].

**Figure 6:**
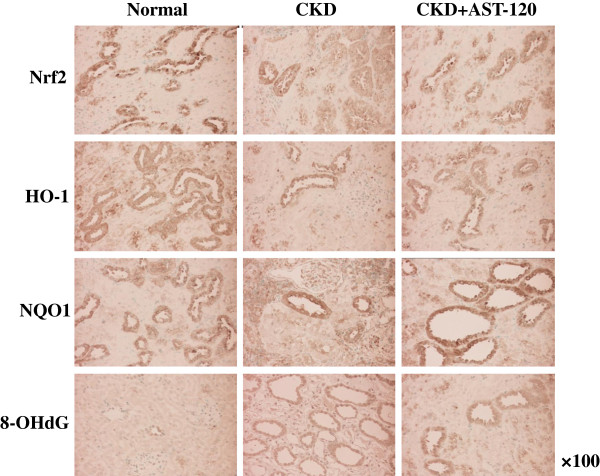
Immunohistochemistry of Nrf2, HO-1, NQO1 and 8-OHdG in the kidneys of normal, CKD, and AST-120-treated CKD rats.

**Figure 7:**
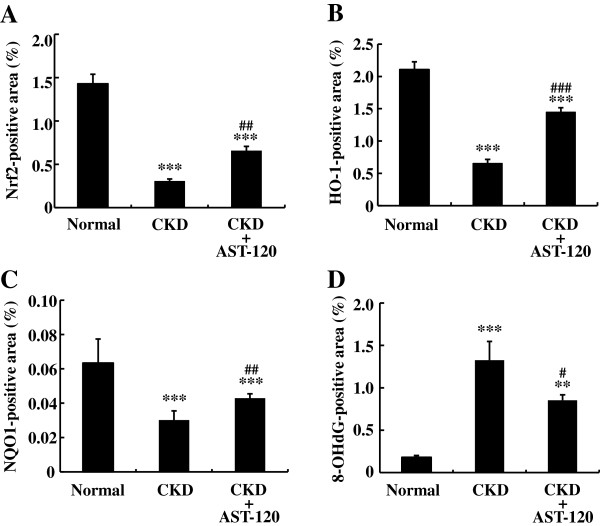
**Immunostaining-positive areas of Nrf2, HO-1, NQO1 and 8-OHdG in the kidneys of normal, CKD, and AST-120-treated CKD rats.** Data are expressed as mean±SE (n=9). **P < 0.01, ***P < 0.001 vs. normal rats; ^#^*P*<0.05, ^##^*P*<0.01, ^###^*P*<0.001 vs. CKD rats.

Nrf2, HO-1, and NQO1 were expressed in tubular epithelial cells, including proximal and distal tubules (Figure 6). In addition, positive staining for 8-OHdG was localized in the nuclei of tubular epithelial cells. AST-120 significantly stimulated Nrf2 expression in the kidneys of CKD rats as compared with control CKD rats, although its expression level was still reduced as compared with normal rats (Figures 6 and 7). Furthermore, AST-120 significantly stimulated renal expression of HO-1 and NQO1 as compared with control CKD rats, although their expression levels were still reduced as compared with normal rats (Figures 6 and 7). The staining level of 8-OHdG was significantly diminished in the kidneys of AST-120-treated CKD rats as compared with CKD rats (Figures 6 and 7). Thus, AST-120 stimulates Nrf2 expression, followed by increased expression of HO-1 and NQO1 and decreased level of ROS in the kidneys of CKD rats as compared with control CKD rats.

## Discussion

The novel findings of the present study are; 1) indoxyl sulfate downregulated expression of Nrf2 in proximal tubular cells, which was alleviated by an inhibitor of NF-κB and siRNA specific to NF-κB p65, 2) indoxyl sulfate downregulated expression of Nrf2, HO-1 and NQO1 and increased expression of 8-OHdG in rat kidneys, and 3) AST-120 which reduced serum indoxyl sulfate, upregulated expression of Nrf2, HO-1, and NQO1 and decreased expression of 8-OHdG in CKD rat kidneys. Thus, the indoxyl sulfate-induced downregulation of Nrf2 leads to the downregulation of HO-1 and NQO1, consequently followed by accumulation of 8-OHdG in the kidneys.

The present study used two animal models. In animal study 1, the effects of indoxyl sulfate administration on Nrf2 expression in the kidneys of not only normal rats but also hypertensive rats were determined, because hypertension is often associated with CKD. In animal study 2, the expression of Nrf2 in the kidney of CKD rats was evaluated as compared with normal rats. Furthermore, the effect of AST-120 on Nrf2 expression in the kidneys of CKD rats was determined, because AST-120 reduces serum indoxyl sulfate level and is clinically used to treat CKD patients in Japan.

The present study first demonstrated that indoxyl sulfate downregulates Nrf2 expression *in vitro* and *in vivo*. As a regulatory mechanism of Nrf2 expression, activated Nrf2 binds to ARE sequence of its own promoter, and thereby upregulates expression of Nrf2 itself [25]. Based on this mechanism, we predict at least two regulatory mechanisms by which indoxyl sulfate suppresses Nrf2 expression. One possibility is that indoxyl sulfate-induced NF-κB activation inhibits Nrf2-ARE pathway because the interaction of p65 with Keap1 promotes reduction of Nrf2 protein level through Nrf2 ubiquitination [26]. Another possibility is that upregulation of p53 expression induced by indoxyl sulfate-induced NF-κB activation is involved in the suppression of Nrf2 mRNA expression, because p53 may bind to ARE sequences and thereby interfere interaction of Nrf2 with ARE sequences [27]. Furthermore, suppression of HO-1 expression may be due to not only downregulation of Nrf2 expression but also binding of p53 to the ARE sequences of HO-1. Because we focused on the crosstalk between indoxyl sulfate and Nrf2, expressions of the NF-κB targets such as Keap1 and p53 are not included in the present study,

The present study first demonstrated that AST-120 alleviates suppression of Nrf2 expression, and thereby induces antioxidative effects in the kidneys of CKD rats. Consequently, AST-120 relieves suppressed expression of HO-1 and NQO1 and attenuates increase of ROS in the CKD rat kidneys. ROS induces necrosis, apoptosis, inflammation, and fibrosis, leading to dysfunction of renal proximal tubular cells. Accumulating evidence indicates that increased humoral factors such as interleukin-6, tumor necrosis factor-α, and angiotensin II induce ROS in CKD rat kidney. Therefore, ROS-induced dysfunction of kidney by these humoral factors also might be suppressed by AST-120 through upregulation of Nrf2 function in the kidney.

Recently, bardoxolone methyl, an activator of Nrf2, has been reported to attenuate progression of CKD and diabetic nephropathy [28,29]. We consider that indoxyl sulfate might contribute to the progression of CKD patients by downregulating renal expression of Nrf2.

## Conclusion

Indoxyl sulfate downregulates renal expression of Nrf2 through activation of NF-κB, followed by downregulation of HO-1 and NQO1 and increased production of ROS. Further, AST-120 upregulates renal expression of Nrf2 in CKD rats by removing serum indoxyl sulfate, followed by upregulation of HO-1 and NQO1 and decreased production of ROS.

## Competing interests

FN is employed by Kureha Corporation. The other authors declare no competing interests.

## Authors’ contribution

DB and MY carried out immunohistochemistry. HS participated in the design of the study, cell culture, quantitative real time PCR, immunoblotting, interpretation of data, and preparation of draft manuscript. FN performed animal experiments. TN conceived of the study, and participated in its design, interpretation of data, and coordination, and helped to draft the manuscript. All authors read and approved the final manuscript.

## Pre-publication history

The pre-publication history for this paper can be accessed here:

http://www.biomedcentral.com/1471-2369/14/56/prepub
